# From Seasonal Strategy to Pandemic Shield: The Case for Prioritizing Influenza Vaccination in Long-Term Care

**DOI:** 10.3390/vaccines13121211

**Published:** 2025-11-30

**Authors:** Jane Barratt, Marco Del Riccio, Stefania Maggi, Jean-Pierre Michel

**Affiliations:** 1 Dr. Jane Barratt Consulting Inc., Toronto, ON M5A 4L1, Canada; 2 Department of Health Sciences, University of Florence, 50134 Florence, Italy; 3 CNR Institute of Neuroscience, 35128 Padova, Italy; 4 Department of Geriatrics and Rehabilitation, University Medical Centre, 1206 Geneva, Switzerland; jean-pierre.michel@unige.ch

**Keywords:** long-term care facilities, influenza vaccination, pandemic preparedness, older adults, health system resilience

## Abstract

Background: The COVID-19 pandemic exposed both the fragility and importance of long-term care facilities (LTCFs). In this context, seasonal influenza vaccination is more than a routine intervention, it is a measurable indicator of system readiness. Methods: We conducted a secondary analysis of the validated 2022 WHO–UNICEF Joint Reporting Form (JRF) on Immunization for all 194 Member States, extracting (i) policy inclusion of older adults and LTCF residents/staff and (ii) availability of numeric coverage data. Findings were interpreted alongside evidence on vaccine effectiveness and delivery in LTCFs as proxies for operational preparedness. Results: Of 194 countries, 128 (66%) reported a national influenza-vaccination policy. Among these, 109 (56%) recommended vaccination for older adults, while only 84 (43%) explicitly included LTCF residents (few countries explicitly named staff). Numeric coverage for older adults was reported by 54 countries (median 55%, range 0–103%), with 13 meeting the WHO ≥75% target. No country reported specific coverage for LTCF residents or staff. Evidence from trials and observational studies shows that vaccination reduces hospitalisation and mortality among residents and that higher staff uptake is associated with fewer resident infections and improved continuity of operations. Facilities achieving high joint coverage appear to reflect stronger governance, supply chains, data systems, and infection-prevention capacity, the same elements required for pandemic response. Conclusion: Influenza vaccination in LTCFs functions as both a barometer and a mechanism of preparedness. Three practical levers should be recognised as core readiness functions: explicit inclusion of LTCF residents and staff in national policy; routine, public reporting of resident and staff coverage; and timely, resourced on-site delivery before seasonal peaks. Embedding these features would better protect those at highest risk and strengthen overall health-system resilience.

## 1. Introduction

The Coronavirus Disease 2019 (COVID-19) pandemic exposed both the fragility and the critical role of long-term care facilities (LTCFs) within health systems. Home to some of the most medically and socially vulnerable populations, these settings became epicentres of morbidity and mortality during the early waves of SARS-CoV-2 [1]. The rapid development and deployment of COVID-19 vaccines demonstrated what can be achieved in crisis but also revealed long-standing weaknesses in seasonal immunisation systems that pre-dated the pandemic [2].

These lessons extend beyond COVID-19. Influenza remains a leading cause of preventable illness and death among older adults, especially in congregate settings [3]. Seasonal influenza vaccination in LTCFs is therefore more than a routine intervention. It is a strategic proxy indicator of health-system readiness, reflecting the capacity to plan, deliver, and document timely vaccination for both residents and staff. Evidence shows that vaccination preserves functional ability, reduces care burden, and supports independence, all of which are central to resilience during health emergencies [4,5].

Thus, LTCFs can be considered sentinel environments; their vaccination coverage mirrors the resilience, responsiveness, and equity of the broader immunisation programme. This article aims to examine how strengthening seasonal influenza vaccination in LTCFs could serve as a cornerstone of pandemic preparedness. It argues that explicit policy inclusion, systematic coverage reporting, and resourced delivery are essential indicators of system readiness.

## 2. Why Long-Term Care Residents Matter in Pandemic Planning

Residents of long-term care facilities (LTCFs) are disproportionately affected by respiratory pathogens such as influenza, respiratory syncytial virus (RSV), and emerging viral infections [6,7]. Their risk is amplified by advanced age, multimorbidity, frailty, and immunosenescence, while shared living spaces accelerate transmission [6,7,8]. During the first year of the COVID-19 pandemic, residents of LTCFs represented an estimated 40–57% of all reported COVID-19-related deaths in many high-income countries, and in some European settings, this proportion exceeded 70% [9]. Across 12 OECD countries, COVID-19 mortality among residents of LTCFs was, on average, more than 20 times higher than among community-dwelling adults aged ≥ 65 years, ranging from 14-fold in Germany to 74-fold in Canada [10]. These figures highlight the extreme vulnerability of congregate-care populations and the consequences of delayed prioritization, fragmented supply chains, and weak integration of LTCFs into national preparedness plans [9]. Although mortality later declined with vaccine roll-out, interventions came too late to prevent catastrophic loss of life [9].

Even before the COVID-19 pandemic, vaccination delivery in long-term care facilities (LTCFs) was fragmented, typically dependent on facility staff or visiting clinicians, with limited coordination across sites and weak integration into public health systems. The pandemic’s first year exposed the operational weaknesses of fragmented systems and, conversely, demonstrated the efficiency gains achievable through coordinated delivery models. In the United States, the Pharmacy Partnership for Long-Term Care Program rapidly mobilised community pharmacies to vaccinate approximately 1.3 million residents and 900,000 staff across more than 12,000 facilities within its first month, achieving 77.8% resident and 37.5% staff first-dose coverage [11,12]. This unprecedented collaboration between public health authorities, pharmacies, and LTCF operators established a functional model for rapid, on-site vaccination at scale. Comparable partnerships in other jurisdictions likewise strengthened supply chains, data systems, and reporting mechanisms, improving both timeliness and equity of vaccine access for residents and staff. These experiences illustrate how embedding LTCFs within national immunisation infrastructure transforms seasonal vaccination from an ad hoc intervention into a reliable preparedness mechanism [13].

Seasonal influenza presents the same vulnerabilities. Influenza outbreaks in LTCFs can propagate rapidly once introduced. A systematic review of 206 outbreaks (including 49 due to influenza) reported a median resident attack rate of 33% (range 4–94%) and a median staff attack rate of 23% (range 3–58%), with a median resident case-fatality rate of 6.5% (range 0–55%) [14]. Globally, seasonal influenza is associated with an estimated 291,243–645,832 respiratory deaths annually, with persons ≥65 years accounting for ~223,000–446,000 deaths (≈70–80% of the total) [15]. Among LTCF residents, influenza is frequently followed by pneumonia, cardiovascular events, and declines in physical and cognitive function—outcomes that compound frailty and increase hospitalisation and mortality [10]. These data underscore the predictable, high-impact burden of influenza in congregate care and the need for systematic vaccination and infection-prevention measures as part of routine preparedness. Facility dynamics amplify these risks: residents live in close quarters, share communal spaces, and often depend on staff for daily activities [16,17]. A single infectious staff member or visitor can initiate transmission within a facility, and once influenza is circulating, control becomes difficult, particularly when staffing levels are already strained. In a cluster-randomised trial of 44 care homes in the United Kingdom, vaccination of staff was associated with a ~40% reduction in resident mortality and a ~50% reduction in hospitalisations during the influenza season [18]. Across reported outbreaks, 7–33% of infected residents require hospitalisation and case-fatality rates can reach up to 55% in severe events [14]. For residents and families, such outbreaks are distressing; for health systems, they drive emergency transfers and excess winter mortality; and for LTC operators, they precipitate staff shortages, reputational harm, and financial penalties.

Despite this predictable and severe burden, a review by the International Federation on Ageing (IFA) of 19 countries found no national immunization strategies with LTCF-specific guidelines [19]. Vaccination demonstrably reduces hospitalization and mortality among residents, while staff immunization lowers introduction and spread [18,20,21].

Protecting LTCF residents is both a clinical and ethical imperative. Older adults in institutional care often cannot advocate for themselves, making guaranteed access to vaccination a matter of justice [22]. With ageing populations driving greater demand for long-term care, integrating LTCFs into vaccination and pandemic planning is not optional but essential.

## 3. Data Source and Analytical Approach

This analysis draws on the 2022 WHO–UNICEF Joint Reporting Form (JRF) on Immunization, the official mechanism through which all 194 Member States report annually on national immunization policies, target groups, and coverage. Data were collected between October 2022 and May 2023, validated by WHO Regional Offices, and cross-checked with national immunization technical advisory group (NITAG) submissions and other official sources [23]. The dataset identifies whether each country has a national influenza vaccination policy, which risk groups are explicitly prioritised (older adults, LTCF residents and staff, health workers, pregnant women, and people with chronic conditions), and, where available, self-reported coverage percentages. Responses are reviewed by WHO staff for completeness and internal consistency before global and regional aggregation.

Analytical Approach. Two variables relevant to pandemic preparedness were extracted: (i) policy inclusion of older adults, LTCF residents and staff, and (ii) availability of numeric coverage data for these groups. These indicators were interpreted as proxies for national readiness and operational capacity in the context of seasonal and pandemic vaccination. No weighting or statistical modelling was applied, and results were summarised descriptively at the global level. Results are reported in Section 4 in direct correspondence with these two indicators.

Transparency Statement. This study uses the same WHO–UNICEF Joint Reporting Form (JRF) dataset described previously [23] but applies an interpretive lens focused on the policy inclusion of long-term care facilities and their relevance to pandemic preparedness.

Limitations. The JRF relies on self-reported national data, which may be incomplete or inconsistently defined across countries. Terms such as “older adult,” “long-term care facility,” and “health-care worker” are not standardised, and some responses reflect policy intent rather than implementation. Coverage data are missing for many Member States or presented in non-numeric form, precluding reliable global estimation. Because subnational or facility-level data are not collected, the analysis cannot capture within-country inequities or data-quality variation. Findings should therefore be interpreted as signals of policy presence and system maturity rather than precise measures of vaccine uptake [23].

## 4. Results: Global Policy and Coverage Signals

Consistent with the two indicators defined in the Methods section, results are presented in two parts: (1) policy inclusion of LTCF residents and staff, and (2) reporting of numeric influenza vaccination coverage data.

Based on the validated 2022 WHO–UNICEF Joint Reporting Form dataset [23], this analysis examines how older adults and residents and staff of long-term care facilities (LTCFs) are prioritised within national influenza-vaccination policies and coverage-reporting systems. Two findings are directly relevant to pandemic preparedness. First, while many countries recommend influenza vaccination for older adults, far fewer explicitly name LTCF residents and staff. Second, even when policy exists, coverage data are inconsistently collected or reported, limiting accountability and obscuring vulnerability in high-risk settings [23].

Policy presence. Among 194 WHO Member States, 128 (66%) reported having a national influenza-vaccination policy [23]. Of these, 109 (56%) recommended vaccination for older adults, while only 84 (43%) explicitly included residents of long-term care facilities in their national guidance. This disparity underscores a persistent omission of congregate-care populations despite their high vulnerability. Regional variation remains marked, with LTCF-specific policies more common in the European Region and the Region of the Americas than in Africa or South-East Asia [23,24,25].

Coverage. Of the 194 Member States, only 54 reported numeric coverage data for older adults through the WHO–UNICEF JRF [23]. Among these, the median coverage was 55% (range 0–103%), and just 13 countries achieved the WHO target of ≥ 75% coverage, a modest improvement from two countries in 2014. No country reported specific coverage data for residents or staff of long-term care facilities, leaving one of the highest-risk populations effectively invisible in global reporting. Where coverage audits do exist, feedback mechanisms are seldom formalised, limiting their value for performance management and quality improvement [19,23].

Facility-level evidence. Facility outcomes are strongly influenced by joint vaccination coverage among residents and staff. Analyses from multiple countries show that higher staff coverage substantially reduces resident morbidity and mortality. In the United States, analysis of 12,364 nursing homes showed that facilities with lower staff vaccination coverage had substantially higher resident COVID-19 case and death rates. Facilities in the lowest quartile of staff vaccination experienced 132% more resident cases and 195% more resident deaths than those in the highest quartile, underscoring the protective value of workforce immunisation [26]. By contrast, a national survey in France found staff influenza-vaccination coverage of approximately 32% in 2018–2019 (ranging from 27% to 43% by staff group), a level associated with increased outbreak frequency [27]. Similar disparities are observed elsewhere: CDC surveillance shows that coverage among long-term-care personnel remains roughly 45%, far below the 95–97% achieved in facilities with mandatory or on-site vaccination policies [28,29].

These findings indicate that seasonal influenza vaccination in long-term care facilities functions as more than a routine public-health measure; it reflects the operational maturity of national immunisation systems. The uneven inclusion of LTCFs in policy, limited coverage reporting, and low workforce uptake reveal gaps in system design and accountability that compromise protection for high-risk populations during health emergencies. In contrast, countries achieving high, jointly monitored coverage among residents and staff demonstrate the governance, data infrastructure, and coordination mechanisms required for effective pandemic response. Collectively, these patterns provide the foundation for interpreting seasonal influenza vaccination as both a barometer and a mechanism of preparedness.

## 5. From Policy to Preparedness: What Seasonal Vaccination Tells Us

Despite inherent data limitations, the 2022 WHO–UNICEF JRF dataset offers valuable insight into the operational readiness of health systems. Seasonal influenza vaccination in long-term care facilities (LTCFs) serves as a practical stress test for the same capacities that determine a country’s pandemic response. When implemented effectively, LTCF vaccination demonstrates that governance, supply chains, workforce capability, data systems, and communication channels are functioning as an integrated whole [30,31]. Conversely, low coverage or delayed delivery exposes the fragility of these systems and the consequences of fragmented accountability [2].

The IFA consensus statement recommends embedding vaccination within infection-prevention and control (IPC) programmes, supported by clear governance and geriatric expertise within national advisory structures [22]. Such integration aligns directly with WHO’s Pandemic Prevention, Preparedness and Response (PPPR) prevention pillar, positioning LTCF vaccination not only as a clinical intervention but as a measurable indicator of system maturity [30,31]. These patterns highlight the strategic value of influenza vaccination in LTCFs as a practical test of system performance. Strengthening its implementation provides insight into, and reinforcement of, the same capacities required for pandemic preparedness.

Evidence of efficacy and effectiveness. It is well recognised that influenza vaccine effectiveness varies by season and by circulating strain. In LTCFs, however, the relevant question is not whether vaccines prevent all infections but whether they reduce severe outcomes and the evidence consistently supports that conclusion. Vaccination lowers the risk of hospitalisation, post-infection disability, complications, and death among older adults [18,20].

Enhanced vaccines further strengthen the case. High-dose and adjuvanted formulations are designed to counter immunosenescence, and both clinical trials and real-world studies demonstrate superior protection in older adults compared with standard-dose vaccines [32,33,34,35,36]. Co-administration with COVID-19 vaccines has been shown to be safe and immunogenic in adults ≥65 years, simplifying delivery during seasonal campaigns [37,38].

For staff, annual influenza vaccination reduces absenteeism and lowers the risk of introducing influenza into the facility, thereby indirectly protecting residents [18,21,39,40]. During COVID-19, facilities with higher staff-vaccine coverage reported fewer resident cases and deaths, underscoring the importance of joint uptake among residents and personnel [26].

Evidence from multi-country analyses and cohort studies confirms the protective impact of vaccination in congregate-care settings. Combined influenza and pneumococcal vaccination is associated with significantly lower all-cause mortality over 12 months, while cohort studies in Hong Kong and mainland China found that dual vaccination in nursing-home residents and older adults reduced mortality and hospitalisations by up to 40% compared with influenza vaccination alone [41,42,43]. Cluster-randomised trials show that high-dose influenza vaccines further reduce respiratory-related hospitalisations compared with standard formulations [33,34].

The benefits of influenza vaccination in LTCFs extend beyond resident health. Vaccination programmes strengthen operational resilience by reducing staff absenteeism during outbreaks and, by preventing influenza-related hospitalisations, help preserve hospital capacity during peak seasons [28,29,30]. Viewed through this operational lens, influenza vaccination is likely cost-efficient. Modest investments in vaccine procurement and on-site delivery offset the substantially higher costs associated with hospital transfers, agency staffing, and outbreak control.

Mapping to preparedness frameworks. The determinants of effective vaccination in long-term care facilities (LTCFs), clear policy, reliable supply chains, workforce readiness, robust data systems, IPC integration, and equitable delivery, closely mirror the pillars of WHO preparedness frameworks. These include the Pandemic Prevention, Preparedness and Response (PPPR) prevention pillar, the International Health Regulations (IHR), and the Health Emergency Preparedness, Response and Resilience (HEPR) framework. In this context, seasonal influenza vaccination in LTCFs functions as both a proxy measure and a practical expression of system preparedness.

Countries that explicitly name LTCFs in their national immunisation policy demonstrate awareness of congregate-care vulnerability and alignment with the PPPR objective of protecting high-risk populations. Reliable vaccine procurement and timely distribution reflect the supply-chain integrity and logistical coordination expected under HEPR, while workforce readiness, supported through trained vaccinators and adequate staffing levels, embodies the IHR requirement for maintaining core health-system capacities. Similarly, the consistent measurement of resident and staff vaccination coverage indicates the presence of functioning data systems and feedback loops essential for real-time situational awareness during public-health emergencies.

Three practical indicators make seasonal LTCF vaccination a useful lens on preparedness: whether LTCFs are explicitly identified in national immunisation policy; whether vaccination coverage for residents and staff is routinely measured and reported; and whether vaccines reach facilities early enough to complete campaigns before the seasonal peak. Tracking these features requires no new infrastructure yet provides a clear signal of whether foundational functions such as governance, logistics, data, and accountability, are working as intended. Regular public reporting of such data would enable benchmarking across jurisdictions and support earlier identification of capacity gaps before the next pandemic stress test.

Implications of the 2024 snapshot. Global data from the 2022 WHO–UNICEF JRF reveal that national influenza vaccination recommendations for older adults are broader than those for residents and staff of LTCFs. This uneven inclusion reflects both the incomplete translation of global guidance into national policy and the limited visibility of congregate-care settings within health-system planning. Coverage data remain inconsistent, with few Member States able to report numeric uptake for older adults and virtually none for LTCF populations. These gaps constrain accountability, obscure vulnerability, and impede targeted intervention.

The absence of LTCF-specific guidance within many national immunisation programmes suggests that system preparedness is still conceived largely through a community- or hospital-centred lens rather than one encompassing institutional care. Where LTCF vaccination frameworks do exist, most notably in parts of Europe and the Americas, they demonstrate that inclusion, measurement, and timely delivery are achievable when governance, supply chains, and data infrastructure are aligned. Conversely, countries without such provisions face predictable implementation barriers: unclear responsibility for vaccine procurement, limited clinical capacity within facilities, and fragmented data systems that prevent monitoring of coverage or outcomes.

The corrective actions implied by these findings are straightforward. Explicitly naming LTCF residents and staff in the national immunisation policy affirms their prioritisation and creates the mandate for operational planning. Establishing standardised, facility-level reporting of vaccination coverage provides the accountability needed to drive performance improvement. Finally, resourcing on-site delivery and integrating vaccination within routine IPC programmes ensures that coverage targets are attainable each season. Taken together, these measures transform seasonal vaccination from a periodic campaign into a structural component of preparedness—one that strengthens governance, reinforces workforce resilience, and protects those most at risk during future health emergencies.

## 6. Policy and Practice Implications

The 2024 findings underscore two interrelated weaknesses in national immunisation systems: the absence of explicit reference to LTCFs and their staff within many vaccination policies, and the lack of consistent mechanisms to monitor coverage across these settings. Addressing these gaps would strengthen both seasonal influenza control and broader pandemic preparedness.

Policy level. Explicit inclusion of LTCF residents and staff in national immunisation schedules creates the necessary policy mandate for prioritisation during seasonal and pandemic vaccination campaigns. Integrating vaccination status into facility quality indicators and linking coverage reporting to electronic health records would enable systematic monitoring. In systems where such measures have been implemented, regular feedback on resident and staff vaccination rates has improved both transparency and accountability.

Operational level. Reliable vaccine supply, on-site delivery, and timely campaign implementation remain central to effective LTCF immunisation. Embedding vaccination within IPC programmes, supported by standing orders and advance procurement, helps ensure readiness before the seasonal peak. Facilities that record vaccination status at admission, use co-administration of indicated vaccines, and monitor cold-chain performance demonstrate higher continuity of protection.

Workforce level. Sustained protection also depends on workforce participation. Evidence shows that staff vaccination reduces influenza-like illness and mortality among residents, while employer-supported delivery, such as free, on-site vaccination on paid time and visible leadership endorsement, achieves substantially higher coverage rates than voluntary or off-site approaches [28,29]. Education initiatives targeting aides and support workers further enhance uptake by linking vaccination to personal and resident safety.

Preparedness dimension. Embedding LTCFs within national pandemic preparedness plans would consolidate these gains. International recommendations emphasise explicit policy inclusion, guaranteed supply, and transparent coverage reporting as markers of system maturity [22]. Viewed through this lens, seasonal influenza vaccination in LTCFs serves not only as a preventive measure but also as an indicator of functional preparedness capacity—where consistent performance reflects strong governance, logistics, and workforce infrastructure.

## 7. Conclusions

Seasonal influenza vaccination in long-term care facilities (LTCFs) is both a measurable barometer of system capacity and a practical mechanism for preparedness. Using the 2022 WHO–UNICEF JRF dataset, we show that although most countries recommend influenza vaccination for older adults, far fewer explicitly name LTCF residents and staff, and numeric coverage reporting remains sparse. Evidence indicates that vaccination reduces severe outcomes for residents and that higher staff uptake improves facility-level protection. Although this is a secondary descriptive analysis based on validated WHO–UNICEF data, it applies a distinct interpretive framework linking influenza vaccination in LTCFs to measurable preparedness capacities—an aspect not explored in previous global analyses.

These findings suggest three observable features, explicit policy inclusion of LTCFs, routine measurement and public reporting of resident and staff coverage, and timely delivery to complete campaigns before seasonal peaks, serve as pragmatic indicators of operational readiness. Although self-reported data and heterogeneous definitions constrain precision, the overall signal is consistent: where these features are present, governance, logistics, data systems, and workforce practices aligned with pandemic response are more likely to be in place. Recognising LTCF influenza vaccination as part of core preparedness capacity can strengthen protection for those at highest risk and enhance overall health-system resilience [44].

## Data Availability

No new data were created in this study. All data referenced are publicly available aggregated indicators from the validated 2022 WHO–UNICEF Joint Reporting Form (JRF) on Immunization and can be accessed through the WHO and UNICEF global immunization data portals.

## References

[1] Andrew M.K., McElhaney J.E., McGeer A., Rockwood K. (2021). COVID-19 in long-term care facilities: Unique challenges and opportunities for research. Lancet Healthy Longev..

[2] World Health Organization (2023). Strengthening Health Emergency Prevention, Preparedness, Response and Resilience.

[3] World Health Organization Vaccinating at Every Age Is Key to Unlocking the Full Potential of Immunization. 2025 Jun 5. https://www.who.int/news/item/05-06-2025-vaccinating-at-every-age-is-key-to-unlocking-the-full-potential-of-immunization.

[4] International Federation on Ageing (2024). Improving Vaccination in Long-Term Care.

[5] Frangos E., Barratt J., Michel J.P., Ecarnot F. (2024). Vaccines in long-term care settings: A narrative review. Gerontology.

[6] Childs A., Zullo A.R., Joyce N.R., McConeghy K.W., van Aalst R., Moyo P., Bosco E., Mor V., Gravenstein S. (2019). The burden of respiratory infections among older adults in long-term care: A systematic review. BMC Geriatr..

[7] Moyo P., Zullo A.R., McConeghy K.W., Bosco E., van Aalst R., Chit A., Gravenstein S. (2020). Risk factors for pneumonia and influenza hospitalizations in long-term care facility residents: A retrospective cohort study. BMC Geriatr..

[8] Mitani S., Ogawara H., Miyagawa S., Doorenbos A.Z., Fukahori H. (2025). Theory development of under what circumstances and what works for promoting disaster preparedness among long-term care facility (LTCF) stakeholders: Protocol for realist review. BMJ Open.

[9] Comas-Herrera A., Zalakaín J., Lemmon E., Henderson D., Litwin C., Hsu A.T., Schmidt A.E., Arling G., Kruse F., Fernández J.-L. (2020). Mortality Associated with COVID-19 Outbreaks in Care Homes: Early International Evidence.

[10] Sepúlveda E.R., Stall N.M., Sinha S.K. (2020). A comparison of COVID-19 mortality rates among long-term care residents in 12 OECD countries. J. Am. Med. Dir. Assoc..

[11] Jones-Jack N., El Kalach R., Yassanye D., Link-Gelles R., Olorukooba A., Demartino A.K., Elam M., Romerhausen D., Vazquez M., Duggar C. (2024). Advancing public health informatics during the COVID-19 pandemic: Lessons learned from a public-private partnership with pharmacies. Vaccine.

[12] Gharpure R., Guo A., Bishnoi C.K., Patel U., Gifford D., Tippins A., Jaffe A., Shulman E., Stone N., Mungai E. (2021). Early COVID-19 first-dose vaccination coverage among residents and staff of skilled nursing facilities—United States, Dec 2020–Jan 2021. MMWR Morb. Mortal. Wkly. Rep..

[13] Stone N.D., Parker Fiebelkorn A., Guo A., Mothershed E., Moccia L., Bell J., Yassanye D., Hall E., Duggar C., Srinivasan A. (2024). Challenges and opportunities during the COVID-19 vaccination efforts in long-term care. Vaccine.

[14] Utsumi M., Makimoto K., Quroshi N., Ashida N. (2010). Types of infectious outbreaks and their impact in elderly care facilities: A review of the literature. Age Ageing.

[15] Iuliano A.D., Roguski K.M., Chang H.H., Muscatello D.J., Palekar R., Tempia S., Cohen C., Gran J.M., Schanzer D., Cowling B.J. (2018). Estimates of global seasonal influenza-associated respiratory mortality: A modelling study. Lancet.

[16] Leece P., Whelan M., Costa A.P., Daneman N., Johnstone J., McGeer A., Rochon P., Schwartz K.L., Brown K.A. (2023). Nursing-home crowding and its association with outbreak-associated respiratory infection in Ontario, Canada (2014–2019): A retrospective cohort study. Lancet Healthy Longev..

[17] van Gulik N., Calder W., Blencowe P., Mikus-Cunningham A., Carmichael R., Bouchoucha S., Kangutkar T., Considine J. (2025). Staff perceptions of their roles in infection prevention and control in residential aged care homes: A qualitative study. Am. J. Infect. Control..

[18] Hayward A.C., Harling R., Wetten S., Johnson A.M., Munro S., Smedley J., Murad S., Watson J.M. (2006). Effectiveness of an influenza vaccine programme for care-home staff to prevent death, morbidity and health-service use among residents: Cluster randomised controlled trial. BMJ.

[19] International Federation on Ageing (2023). Improving Adult Vaccination Policy in Long-Term Care Settings: IFA Technical Report.

[20] Darvishian M., van den Heuvel E.R., Bissielo A., Castilla J., Cohen C., Englund H., Gefenaite G., Huang W.-T., la Bastide-van Gemert S., Martinez-Baz I. (2017). Effectiveness of seasonal influenza vaccination in community-dwelling elderly people: An individual participant data meta-analysis. Lancet Respir. Med..

[21] Lemaitre M., Meret T., Rothan-Tondeur M., Belmin J., Lejonc J.L., Luquel L., Piette F., Salom M., Verny M., Vetel J.-M. (2009). Effect of influenza vaccination of nursing-home staff on mortality of residents: A cluster-randomised trial. J. Am. Geriatr. Soc..

[22] International Federation on Ageing (2024). Mobilizing Policy Action–Prioritizing Vaccination in Long-Term Care: Consensus Statement and Recommendations.

[23] Goldin S., Brooks D., Jorgensen P., Wijesinghe P., Cho H., Attia R., Doshi R., Nogareda F., Herring B., Dumolard L. (2024). Seasonal influenza vaccination: A global review of national policies in 194 WHO Member States in 2022. Vaccine.

[24] European Centre for Disease Prevention and Control (ECDC) (2024). Survey Report on National Seasonal Influenza Vaccination Recommendations and Coverage Rates in EU/EEA Countries.

[25] Bechini A., Boccalini S., Del Riccio M., Pattyn J., Hendrickx G., Wyndham-Thomas C., Gabutti G., Maggi S., Ricciardi W., Rizzo C. (2024). Overview of adult immunization in Italy: Successes, lessons learned and the way forward. Hum. Vaccin. Immunother..

[26] McGarry B.E., Gandhi A.D., Grabowski D.C., Barnett M.L. (2022). Nursing-home staff vaccination and COVID-19 outcomes. N. Engl. J. Med..

[27] Vaux S., Fonteneau L., Venier A.G., Gautier A., Soing Altrach S., Parneix P., Lévy-Bruhl D. (2022). Influenza vaccination coverage of professionals working in nursing homes in France, 2018–2019 season: A cross-sectional survey. BMC Public Health.

[28] Bell J., Meng L., Barbre K., Wong E., Lape-Newman B., Koech W., Soe M.M., Woods A., Kuhar D.T., Stuckey M.J. (2024). Influenza and COVID-19 vaccination coverage among health-care personnel—United States, 2023–2024 respiratory virus season. MMWR Morb. Mortal. Wkly. Rep..

[29] Black C.L., Yue X., Ball S.W., Fink R.V., de Perio M.A., Laney A.S., Williams W.W., Graitcer S.B., Fiebelkorn A.P., Lu P.J. (2017). Influenza vaccination coverage among health-care personnel—United States, 2016–2017 influenza season. MMWR Morb. Mortal. Wkly. Rep..

[30] Ashby E., Jefferson K.M.P., Yadav P., Anupindi R., National Academy of Medicine, National Academies of Sciences, Engineering, and Medicine, Health and Medicine Division, Board on Global Health (2021). Committee on Addressing Issues of Vaccine Distribution and Supply Chains to Advance Pandemic and Seasonal Influenza Preparedness and Response. Globally Resilient Supply Chains for Seasonal and Pandemic Influenza Vaccines.

[31] Public Health Ontario Best Practices. https://www.publichealthontario.ca/en/Health-Topics/Infection-Prevention-Control/Best-Practices-IPAC.

[32] McConeghy K.W., Davidson H.E., Canaday D.H., Han L., Saade E., Mor V., Gravenstein S. (2021). Cluster-randomized trial of adjuvanted versus non-adjuvanted trivalent influenza vaccine in 823 US nursing homes. Clin. Infect. Dis..

[33] Gravenstein S., Davidson H.E., Taljaard M., Ogarek J., Gozalo P., Han L., Mor V. (2017). Comparative effectiveness of high-dose vs standard-dose influenza vaccination on hospital admissions among US nursing-home residents: Cluster-randomised trial. Lancet Respir. Med..

[34] Johansen N.D., Modin D., Loiacono M.M., Harris R.C., Dufournet M., Larsen C.S., Larsen L., Wiese L., Dalager-Pedersen M., Claggett B.L. (2025). High-dose influenza vaccine effectiveness against hospitalisation in older adults. N. Engl. J. Med..

[35] Pardo-Seco J., Rodríguez-Tenreiro-Sánchez C., Giné-Vázquez I., Mallah N., Mirás-Carballal S., Piñeiro-Sotelo M., Cribeiro-González M., Conde-Pájaro M., González-Pérez J.M., Rivero-Calle I. (2025). High-dose influenza vaccine to reduce hospitalisations. N. Engl. J. Med..

[36] Smith C.L., Bednarchik B., Aung H., Wilk D.J., Boxer R.S., Daddato A.E., Wilson B.M., Gravenstein S., Canaday D.H. (2023). Humoral and cellular immunity induced by adjuvanted and standard trivalent influenza vaccine in older nursing-home residents. J. Infect. Dis..

[37] Izikson R., Brune D., Bolduc J.S., Bourron P., Fournier M., Moore T., Pandey A., Perez L., Sater N., Shrestha A. (2022). Safety and immunogenicity of a high-dose quadrivalent influenza vaccine administered with a third dose of mRNA-1273 SARS-CoV-2 vaccine in adults ≥ 65 years. Lancet Respir. Med..

[38] McGrath L.J., Malhotra D., Miles A.C., Welch V.L., Di Fusco M., Surinach A., Barthel A., Alfred T., Jodar L., McLaughlin J.M. (2023). Estimated effectiveness of co-administration of the BNT162b2 BA.4/5 COVID-19 vaccine with influenza vaccine. JAMA Netw. Open.

[39] Li T., Qi X., Li Q., Tang W., Su K., Jia M., Yang W., Xia Y., Xiong Y., Qi L. (2021). A systematic review and meta-analysis of seasonal influenza vaccination of health workers. Vaccines.

[40] Antinolfi F., Battistella C., Brunelli L., Malacarne F., Bucci F.G., Celotto D., Cocconi R., Brusaferro S. (2020). Absences from work among health-care workers: Are they related to influenza shot adherence?. BMC Health Serv. Res..

[41] Chan T.C., Hung I.F., Luk J.K., Shea Y.F., Chan F.H., Woo P.C., Chu L.W. (2012). Prevention of mortality and pneumonia among nursing-home older adults by dual pneumococcal and seasonal influenza vaccination. J. Am. Med. Dir. Assoc..

[42] Jiang Y., Ye Z., Chen D., Shu Y. (2020). Dual influenza and pneumococcal vaccination associated with lower risks of hospitalisation among elderly adults in China: A retrospective cohort study. Emerg. Microbes Infect..

[43] Zhang Y.Y., Tang X.F., Du C.H., Wang B.B., Bi Z.W., Dong B.R. (2016). Comparison of dual influenza and pneumococcal polysaccharide vaccination with influenza alone for preventing pneumonia and reducing mortality among the elderly: A meta-analysis. Hum. Vaccines Immunother..

[44] Centers for Disease Control and Prevention (2023). Influenza Vaccination: A Summary for Clinicians.

